# Preclinical characterization of a non-peptidomimetic HIV protease inhibitor with improved metabolic stability

**DOI:** 10.1128/aac.01373-23

**Published:** 2024-02-21

**Authors:** Andrew Mulato, Eric Lansdon, Ron Aoyama, Johannes Voigt, Michael Lee, Albert Liclican, Gary Lee, Eric Singer, Brian Stafford, Ruoyu Gong, Bernard Murray, Julie Chan, Johnny Lee, Yili Xu, Shekeba Ahmadyar, Ana Gonzalez, Aesop Cho, George J. Stepan, Uli Schmitz, Brian Schultz, Bruno Marchand, Boris Brumshtein, Ruth Wang, Helen Yu, Tomas Cihlar, Lianhong Xu, Stephen R. Yant

**Affiliations:** 1 Department of Virology, Gilead Sciences, Foster City, California, USA; 2 Department of Structural Biology and Chemistry, Gilead Sciences, Foster City, California, USA; 3 Department of Drug Metabolism, Gilead Sciences, Foster City, California, USA; 4 Department of Discovery Sciences and Technology, Gilead Sciences, Foster City, California, USA; 5 Department of Medicinal Chemistry, Gilead Sciences, Foster City, California, USA; IrsiCaixa Institut de Recerca de la Sida, Barcelona, Spain

**Keywords:** HIV, protease, antiviral agents, antiviral pharmacology, antiretroviral resistance, pharmacokinetics, preclinical drug studies, drug discovery

## Abstract

Protease inhibitors (PIs) remain an important component of antiretroviral therapy for the treatment of HIV-1 infection due to their high genetic barrier to resistance development. Nevertheless, the two most commonly prescribed HIV PIs, atazanavir and darunavir, still require co-administration with a pharmacokinetic boosting agent to maintain sufficient drug plasma levels which can lead to undesirable drug-drug interactions. Herein, we describe GS-9770, a novel investigational non-peptidomimetic HIV PI with unboosted once-daily oral dosing potential due to improvements in its metabolic stability and its pharmacokinetic properties in preclinical animal species. This compound demonstrates potent inhibitory activity and high on-target selectivity for recombinant HIV-1 protease versus other aspartic proteases tested. In cell culture, GS-9770 inhibits Gag polyprotein cleavage and shows nanomolar anti-HIV-1 potency in primary human cells permissive to HIV-1 infection and against a broad range of HIV subtypes. GS-9770 demonstrates an improved resistance profile against a panel of patient-derived HIV-1 isolates with resistance to atazanavir and darunavir. In resistance selection experiments, GS-9770 prevented the emergence of breakthrough HIV-1 variants at all fixed drug concentrations tested and required multiple protease substitutions to enable outgrowth of virus exposed to escalating concentrations of GS-9770. This compound also remained fully active against viruses resistant to drugs from other antiviral classes and showed no *in vitro* antagonism when combined pairwise with drugs from other antiretroviral classes. Collectively, these preclinical data identify GS-9770 as a potent, non-peptidomimetic once-daily oral HIV PI with potential to overcome the persistent requirement for pharmacological boosting with this class of antiretroviral agents.

## INTRODUCTION

HIV-1 infection is a global epidemic with an estimated 38.4 million people living with HIV (PLWH) and 1.5 million new infections in 2021 (1). Although the successful development and use of antiviral drugs to treat HIV-1 infection has resulted in a significant reduction in mortality, it requires lifelong therapy with a combination of antiretroviral agents (ARVs) to keep the virus suppressed. Currently approved ARVs target one or more essential steps in the HIV replication cycle and include the inhibition of reverse transcription by nucleoside and non-nucleoside reverse transcriptase inhibitors (NRTIs, NNRTIs), the inhibition of multiple essential capsid functions (nuclear import and disassembly of the viral core, virion production, and proper capsid formation) by a capsid inhibitor (CAI), the inhibition of viral DNA integration by integrase strand transfer inhibitors (INSTIs), and the inhibition of HIV Gag and Gag-Pol polyprotein cleavage by protease inhibitors (PIs).

The active HIV-1 viral protease (PR) is a homodimer with 99 residues per monomer and belongs to a larger class of proteolytic enzymes known as aspartic proteases (also called aspartyl or aspartate proteases). These enzymes contain an active site with two aspartic acid (D) residues, one from each monomer, essential for coordinating peptide-bond cleavage. This class of enzymes includes viral proteases such as those encoded by HIV-1 and HIV-2 as well as various host cell proteases including those expressed in humans such as β-amyloid precursor protein cleaving enzyme 1 (BACE1), pepsin, cathepsin D and E, napsin, and renin (2). The HIV-1 protease is one of three essential enzymes encoded by the viral Gag-Pol polyprotein—the others being reverse transcriptase (RT) and integrase (IN)—and becomes catalytically active when Gag-Pol subunits sequester at the inner cell membrane and eventually dimerize during virion assembly and release from cells. Activation of the viral protease enables rapid coordinated cleavage of immature Gag and Gag-Pol precursor polyproteins into their fully cleaved and mature active forms, an essential process for proper virion maturation and formation of an infectious virion (3, 4).

HIV protease remained a popular target of antiviral drug discovery efforts for several decades, resulting in 10 novel compounds approved by the FDA for the treatment of HIV-1 infection in PLWH. These compounds include saquinavir (SQV), the first approved PI in 1995, followed by indinavir (IDV, 1996), ritonavir (RTV, 1996), nelfinavir (NFV, 1997), amprenavir (APV, 1999), lopinavir (LPV, 2000), tipranavir (TPV, 2003), fosamprenavir (FPV, 2003), atazanavir (ATV, 2003), and finally darunavir (DRV, 2006). All but one of these inhibitors, TPV, have a peptide-like core and, unlike the once daily antiviral agents associated with standard-of-care in PLWH, all current PIs still require multiple doses each day.

To help meet the less-frequent dosing preferences of PLWH, the two most recently approved PIs, ATV and DRV, are each most commonly co-administered with a pharmacokinetic (PK) enhancing agent commonly referred to as a booster, such as ritonavir or cobicistat, to block their metabolism by CYP3A enzymes and thereby extend their plasma half-life *in vivo* to enable once-daily dosing (5, 6). In these cases, inhibition of a key component of drug metabolism *via* the use of a PK boosting agent also increases the potential for undesirable drug-drug interactions (DDIs) in PLWH since these individuals are likely to take other medications metabolized through the same pathway at some point in their lives. Treatment guidelines from the Department of Health and Human Services (DHHS) and World Health Organization (WHO) recommend boosted PIs as part of a preferred second-line antiretroviral regimen and yet there has been no new PI approved by the FDA in over 17 years. To date, the requirement for PK boosting with this class of ARV to achieve once-daily dosing comparable to current standard-of-care and resulting DDIs have not been resolved (7, 8). Ideally, progress toward a new HIV PI would require potent activity against various subtypes and prevalent drug-resistant variants without the need for pharmacokinetic enhancement.

Recent progress in PI design was made against another aspartyl protease, BACE1, where orally bioavailable brain penetrating small-molecule inhibitors based on a novel, non-peptidomimetic scaffold were discovered (9). These inhibitors contain a guanidine or amidine moiety which forms a hydrogen bonding network with the aspartic acid residues in the enzyme’s active site (10). Importantly, these BACE1 inhibitors do not require PK boosting and have the potential for once daily oral dosing. Inspired by this discovery, we set out to identify a potent HIV PI with a guanidine or amidine core and utilized structure-based drug design to progressively identify ligands that could adequately fill the enzyme’s substrate binding pockets.

In this work, we report the preclinical characterization of GS-9770, a novel non-peptidomimetic HIV PI with favorable pharmacological and metabolic profiles supportive of unboosted once-daily oral administration potential. GS-9770 demonstrates a favorable resistance profile and potent and selective antiviral activity against various HIV subtypes and viral isolates with resistance to DRV and ATV, with minimal cytotoxicity observed in multiple human cell types. Two-drug combination studies and cross-resistance studies also suggest that GS-9770 is compatible with drugs from other classes of HIV antivirals.

## RESULTS

### Structure-enabled discovery of GS-9770, a non-peptidomimetic HIV PI

**Fig 1:**
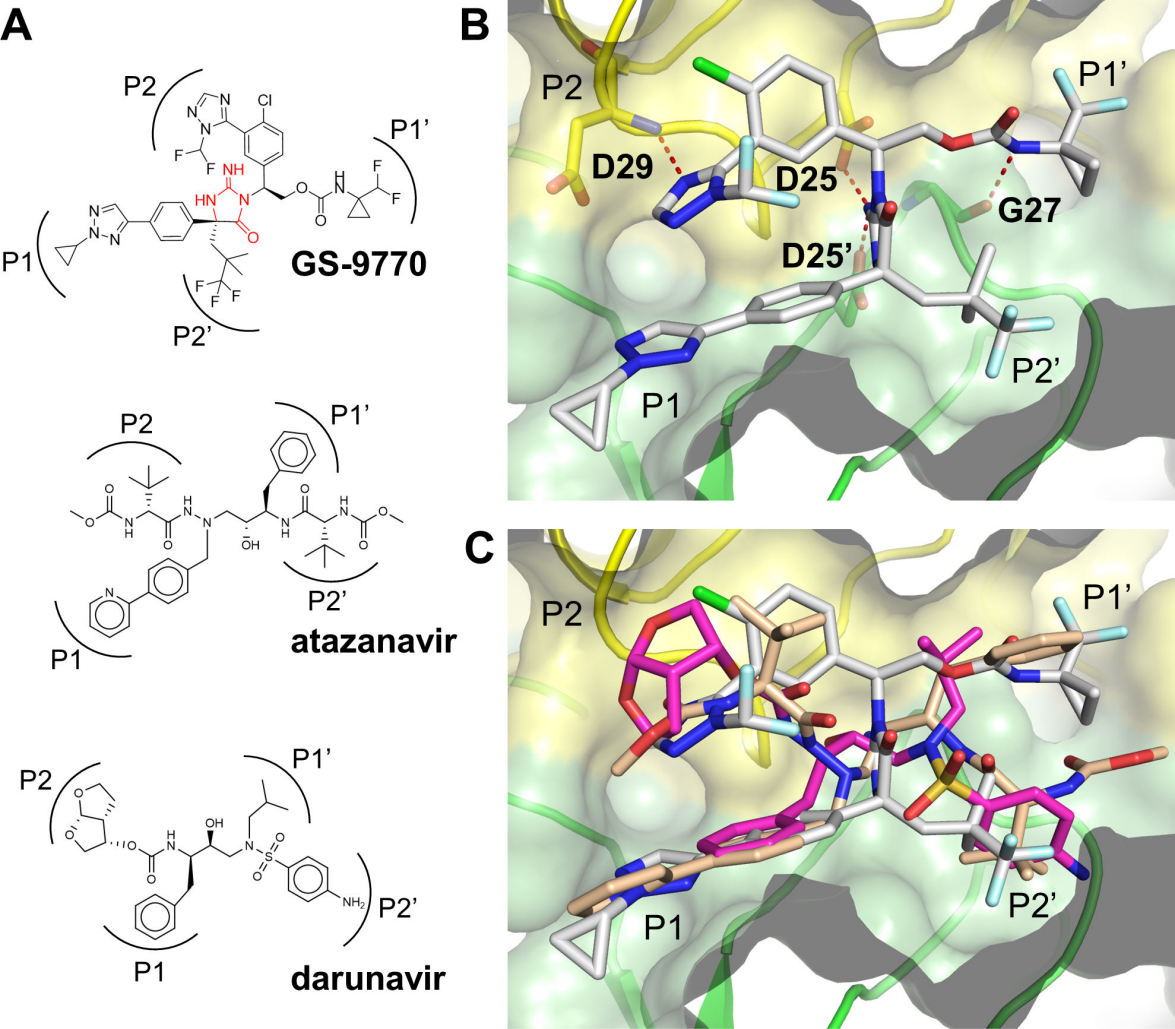
X-ray crystal structure of GS-9770 bound to HIV-1 protease. (**A**) Chemical structure of GS-9770 and two FDA-approved protease inhibitors, atazanavir (ATV) and darunavir (DRV), with the corresponding substrate binding pockets for each inhibitor side-chain indicated as P1, P2, P1′, and P2′. The iminohydantoin core of GS-9770 is highlighted in red. (**B**) X-ray crystal structure of GS-9770 bound to HIV-1 protease with the homodimer subunits shaded yellow and green. The essential catalytic aspartic acid (**D**) residues are labeled D25 and D25′. Hydrogen bond interactions between GS-9770 and the catalytic residues, as well as D29 in the P2 pocket and G27 in the P1′ pocket, are shown as dashed red lines. (**C**) X-ray crystal structures of GS-9770 (grey), ATV (tan), and DRV (magenta) overlayed on recombinant HIV-1 protease to demonstrate similar binding with all four substrate binding pockets occupied by the three compounds.

Current clinically approved PIs such as ATV and DRV contain a peptidomimetic backbone and are co-dosed with pharmacokinetic boosters such as ritonavir or cobicistat to maintain therapeutic plasma drug levels. In an attempt to identify new PIs without the intrinsic liabilities associated with a peptide backbone, a structure-based drug design campaign was performed in which iminohydantoin and other cyclic amidine/guanidine fragments were initially docked to a DRV-bound HIV protease crystal structure (PDB:4DQB) and then suitable substituents for each binding pocket were identified through molecular docking. These structure-based modeling efforts, when coupled with innovative medicinal chemistry and rigorous biochemical, cellular, and metabolic profiling of all new compounds, resulted in the discovery of GS-9770, a potent, investigational HIV PI with an iminohydantoin core (Fig. 1A).

To better understand the interaction between HIV-1 protease and GS-9770, the protein was co-crystallized with GS-9770 and the X-ray crystal structure determined at 1.3 Å resolution (Fig. 1B; Table S1). The iminohydantoin moiety of GS-9770 engages the essential catalytic amino acid residue D25 in each monomer of the HIV-1 PR homodimer. Additional hydrogen bonds are formed in the P2 pocket between the imidazole and the backbone nitrogen of D29 as well as the P1′ carbamate to the backbone carbonyl of G27. The primary interactions observed in the P1 and P2′ pockets are hydrophobic in nature. Molecular docking of GS-9770 to the active site of protease showed a similar binding mode as the PIs atazanavir and darunavir with each of the four substrate binding pockets occupied by GS-9770 (Fig. 1C).

### GS-9770 mechanism of action

**Fig 2:**
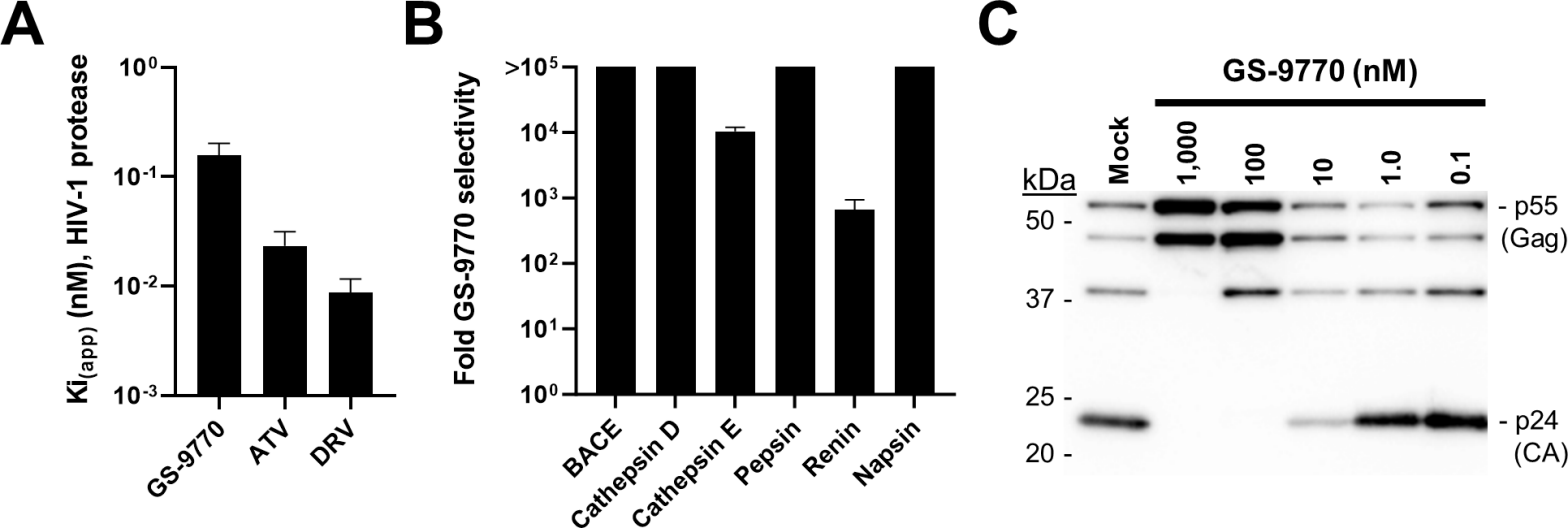
GS-9770 mechanism of action. (**A**) Apparent inhibitory constant (Ki(app)) of GS-9770, atazanavir (ATV), and darunavir (DRV) against wild-type recombinant HIV-1 protease. Data are mean ± s.d. values from at least four independent experiments each performed in duplicate. (**B**) Selectivity of GS-9770 for HIV-1 protease compared to non-HIV aspartic proteases. Fold GS-9770 selectivity calculated by dividing its mean half-maximal inhibitory concentration (IC_50_) value for each non-HIV enzyme by the Ki(app) value for HIV-1 protease. For non-HIV enzymes, the IC_50_ is significantly higher than the enzyme concentration used and is considered equal to Ki(app), and thus the selectivity values reflect true ratios of binding affinities. Data are from at least three independent experiments performed in quadruplicate. (**C**) Western blot showing dose-dependent inhibition of Gag (**P55**) polyprotein cleavage by GS-9770 in HEK293T cells transfected with an HIV-1 expression plasmid.

To investigate the inhibitory activity and specificity of GS-9770 for HIV-1 protease, we began by performing fluorogenic biochemical enzyme inhibition assays utilizing purified recombinant HIV-1 PR and six unrelated aspartic proteases. GS-9770 demonstrated an apparent inhibitory constant (*K*_i(app)_) value of 0.16 nM against HIV-1 PR as compared to 0.023 and 0.009 nM for the control PIs atazanavir and darunavir, respectively (Fig. 2A). The inhibitory activity of GS-9770 against HIV-1 protease was much more pronounced than was observed for each of six unrelated aspartic proteases tested (BACE, cathepsin D, cathepsin E, pepsin, renin, and napsin). For these non-HIV-1 proteases, the *K*_i(app)_ values for GS-9770 ranged from 106 nM to >100 µM, resulting in fold-selectivity values for GS-9770 against HIV-1 protease over these off-target aspartic proteases that ranged from 662 to >710,000 (Fig. 2B).

To assess whether GS-9770 also inhibited HIV-1 protease activity in cells, we measured its effect on the intracellular processing of HIV-1 Gag (p55) polyprotein (NL4.3 strain) by the viral protease. Lysates from virus-producing HEK293T cells cultured in the absence and presence of varying concentrations of GS-9770 were prepared 2 days post-transfection and analyzed by anti-p24 immunoblotting against capsid and its precursor polyproteins (Fig. 2C). Consistent with its low inhibitory constant for HIV-1 protease, GS-9770 substantially reduced Gag polyprotein cleavage and the concomitant release of mature p24 protein at concentrations equal to 10 nM, with complete inhibition of p24 formation at 100 nM.

### Metabolic stability of GS-9770

To determine whether this new iminohydantoin-based HIV PI provided any potential metabolic upside relative to existing peptidomimetic PIs, GS-9770 and two control HIV PIs were independently incubated in the presence of human liver microsomes and the amount of parent compound remaining after different incubation times determined by liquid chromatography-tandem mass spectrometry (LC-MS/MS) analysis (Table S2). The predicted percent hepatic extraction (Eh%), half-life (T_1/2_), and predicted human clearance (CL) values for GS-9770 were 9%, >395 minutes, and 0.11 L/h/kg, respectively. In comparison, ATV and DRV exhibited 4- to 5-fold higher hepatic extraction (Eh% ranging from 63% to 81%), >17- to >56-fold lower metabolic stability (T_1/2_ ranging from 7 to 24 minutes) and 7- to 10-fold higher clearance values (CL ranging from 0.8 to 1.1 L/h/kg) relative to that of GS-9770, respectively. A subsequent more precise determination of metabolic stability was performed using radiolabeled GS-9770 incubated in the presence of human liver microsomes and produced a predicted human plasma clearance value of 0.09 L/h/kg for this compound.

To assess whether the improved metabolic stability observed *in vitro* translated *in vivo*, the pharmacokinetic profile of GS-9770 was evaluated in three preclinical species following intravenous (IV) and oral (PO) dosing (Table 1). GS-9770 was well tolerated at the doses tested (1 mg/kg IV, 4–5 mg/kg PO) and demonstrated high oral bioavailability (%F), ranging from 49% in rats to 100% in dogs and cynomolgus monkeys. The terminal half-life for GS-9770 in rat, dog, and cynomolgus monkeys was 10.6, 6.7, and 7.4 hours, respectively, with corresponding plasma clearance (CL) values of 0.21, 1.13, and 0.34 L/h/kg. These *in vivo* plasma CL values for GS-9770 aligned well with the *in vitro* predicted blood CL values of 0.07, 0.72, and 0.15 L/h/kg measured in liver microsome fractions from rat, dog, and cynomolgus monkey, respectively.

**TABLE 1:** Summary of pharmacokinetic parameters for GS-9770 in nonclinical species

Parameter^*a*^	Sprague-Dawley rat^*b*^^,*c*,*d*^	Beagle dog^*b*,*e*,*d*^	Cynomolgus monkey^*f*^^,*e*,*d*^
AUC_0–24h_ (µM·h)	4,910 ± 455	916 ± 207	2,210 ± 907
CL (L/h/kg)	0.21 ± 0.02	1.1 ± 0.3	0.34 ± 0.1
V_ss_ (L/kg)	2.7 ± 0.3	5.0 ± 0.5	2.6 ± 0.8
Terminal t_1/2_ (h)	10.6 ± 1.4	6.7 ± 0.6	7.4 ± 0.2
F%	45.9 ± 9.8	100 ± 20	100 ± 34

^*a*^ AUC_0–24 h_ = area under curve from 0 to 24 hours; CL = drug clearance; V_ss_ = volume of distribution; F% = oral bioavailability.

^*b*^ Dosed as 1 mg/kg IV and 5 mg/kg PO.

^*c*^ IV dose formulated as a solution in 5% ethanol, 40% PEG300, and 55% water (pH 2.0).

^*d*^ Values represent mean ± s.d. values obtained from three individual animals.

^*e*^ IV dose formulated as a solution in 5% ethanol, 40% PEG300, and 55% water (pH 2.5–2.8).

^*f*^ Dosed as 1 mg/kg IV and 4 mg/kg PO.

### Antiviral activity and selectivity of GS-9770

In a 5-day cytoprotection assay utilizing MT-4 cells acutely infected with the syncytia-inducing HIV-1 IIIb strain, GS-9770 showed high antiviral potency identical to that of atazanavir (ATV, a control PI), with a mean 50% effective concentration (EC_50_) value of 11 nM for each compound (Table 2). In parallel studies, GS-9770 and ATV each exhibited low cytotoxicity in uninfected MT-4 cells, with a mean 50% cytotoxic concentration (CC_50_) values of 6.6 and >50 µM, respectively, resulting in corresponding selectivity index (CC_50_/EC_50_ ratio) values of 620 for GS-9770 and 4,700 for ATV in this T-cell line. When tested in primary human immune target cells acutely infected with HIV-1 BaL strain using a virus (p24) production assay, GS-9770 exhibited mean EC_50_ and selectivity index values of 8.5 nM and 1,100 in CD4 +T lymphocytes and 11 nM and 1,500 in monocyte-derived macrophages, respectively. The antiviral activity and cytotoxicity values for the control PI ATV in these primary target cells did not differ appreciably from those observed in the MT-4 cell line.

**TABLE 2:** Anti-HIV-1 potency, cytotoxicity, and selectivity in human cells

Compound^*a*^	MT-4 T cell line			CD4^+^ T-lymphocytes			Monocyte-derived macrophages		
	EC_50_ (nM)^*b*^	CC_50_ (µM)^*b*^	SI^*c*^	EC_50_ (nM)^*d*^	CC_50_ (µM)^*d*^	SI^*c*^	EC_50_ (nM)^*d*^	CC_50_ (µM)^*d*^	SI
GS-9770	11 ± 2	6.6 ± 1.3	620	8.5 ± 2.5	9.0 ± 0.4	1,100	11 ± 14	16 ± 3	1,500
ATV	11 ± 3	51 ± 8	4,700	4.3 ± 1.9	>50	>12,000	16 ± 12	>50	>3,100

^*a*^ ATV = atazanavir (PI).

^*b*^ EC_50_ (HIV-1_IIIb_ infected) and CC_50_ (mock infected) values represent the geomean (± s.d.) obtained from at least 13 experiments performed in quadruplicate.

^*c*^ SI = selectivity index (CC_50_/EC_50_ ratio.

^*d*^ EC_50_ (HIV-1_BaL_ infected) and CC_50_ (mock infected) values represent the mean (± s.d.) values obtained from three independent experiments performed in triplicate.

To determine whether or not the antiviral activity of GS-9770 was specific for HIV, we measured its antiviral activity against a panel of non-HIV RNA and DNA viruses. Although GS-9770 exhibited weak antiviral activity against markers of hepatitis B virus (HBV) in a hepatocellular carcinoma cell line overexpressing the HBV receptor sodium taurocholate co-transporting polypeptide (HepG2-NTCP), hepatitis C virus (HCV) in Huh7-Lunet genotype 1b and 2 a replicon cells, respiratory syncytial virus (RSV) in a normal human bronchial epithelial (NHBE) cell line, and severe acute respiratory syndrome coronavirus 2 (SARS-CoV-2) in a lung alveolar cell line stably overexpressing the SARS-CoV-2 receptor human angiotensin I converting enzyme 2 (A549-ACE2) (EC_50_ range of 2.5 to 34 µM), it displayed comparable cytotoxicity in each of the corresponding cell lines (CC_50_ range of 4.7 to 15.1 µM). Accordingly, GS-9770 showed a maximal selectivity index (e.g., CC_50_/EC_50_ ratio) value of 1.9 indicating that this compound is not a selective inhibitor of these non-HIV viruses (Table S3).

### Cytotoxicity of GS-9770 in human cell lines and primary cells

The cytotoxicity of GS-9770 was further assessed in five non-HIV-1 permissive human cell lines, including a prostate carcinoma cell line (Gal-PC3), three hepatoma cell lines (HEp-2, Huh7, and Gal-HepG2), and an embryonic lung fibroblast cell line (MRC-5). The Gal-HepG2 and Gal-PC3 cells used in this study were galactose-adapted to enhance sensitivity against mitochondrial toxicity (Marroquin, et al. 2007). GS-9770 displayed comparable or reduced cytotoxicity in each of these five human cell lines relative to the MT-4 T cell line, with the mean CC_50_ values ranging from 13 to 20 µM (Table S4). The cytotoxicity of GS-9770 was also evaluated in primary human hepatocytes and PBMCs (both stimulated and unstimulated) obtained from each of three independent donors. The mean CC_50_ values for GS-9770 in these primary human cells were comparable to those observed in human cell lines and ranged from 9.1 to 34 µM (Table S4). Across all of the cell types tested, the mean CC_50_ values for the positive control compound puromycin (a protein synthesis inhibitor) ranged from 0.31 to 1.6 µM when assayed in parallel.

### Antiviral activity of GS-9770 against a panel of diverse HIV-1 and HIV-2 clinical isolates

**Fig 3:**
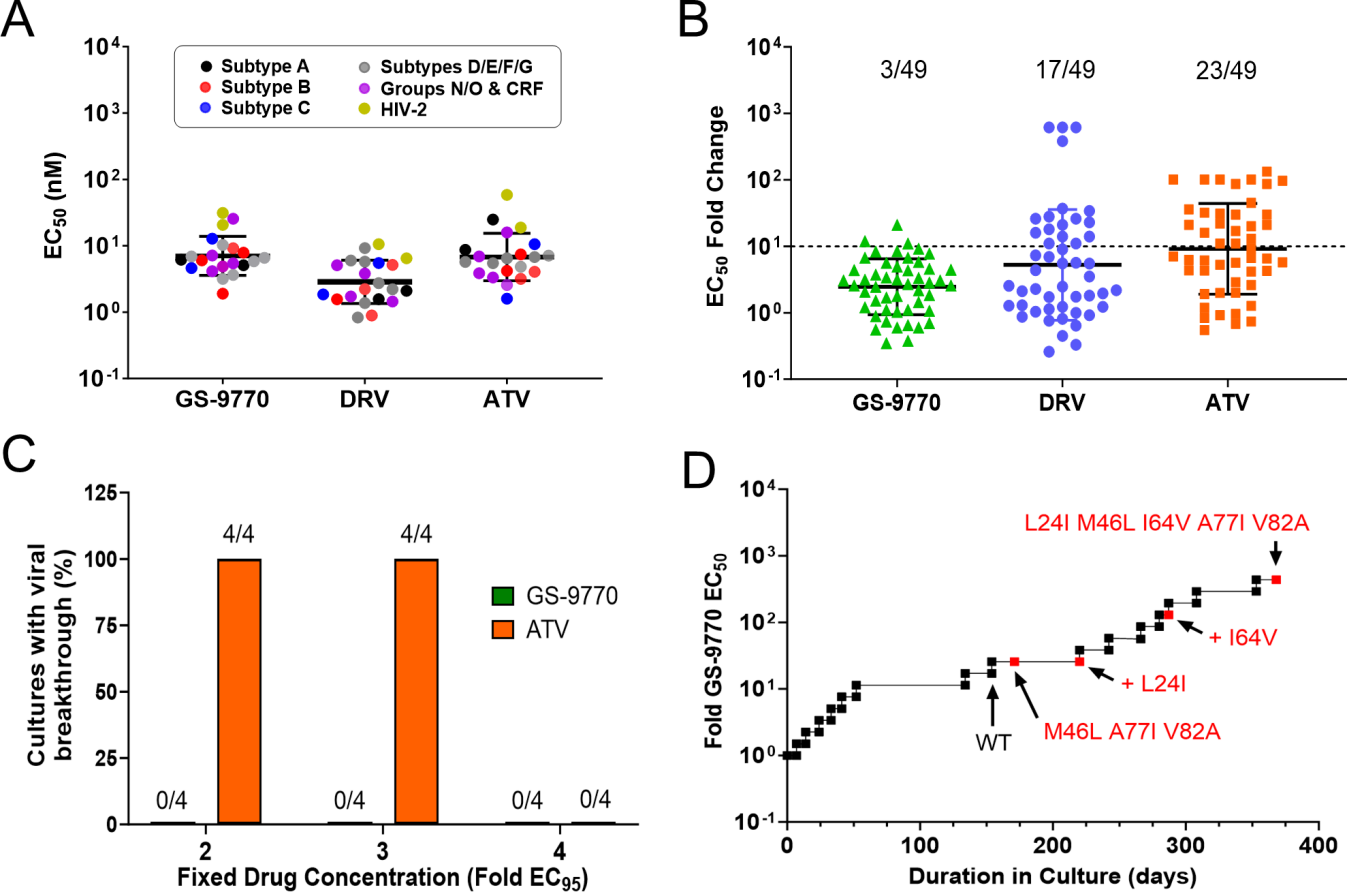
Activity of GS-9770 in HIV-infected cells. (**A**) Inhibition of 20 HIV-1 and 2 HIV-2 clinical isolates in human PBMCs by GS-9770, darunavir (DRV), and atazanavir (ATV). Symbols represent individual isolates assayed in triplicate cell cultures. Center line and error bars represent geometric mean ± s.d. values across all viruses tested for each inhibitor. (**B**) Results of the PhenoSense assay performed at Monogram Biosciences against a panel of 49 patient-derived reporter viruses with PI resistance-associated substitutions in the protease gene. Data represent fold change in IC_50_ for each patient isolate. Bars represent geometric means of 2.5, 5.3, and 9.1 for GS-9770, DRV, and ATV, respectively, with error bars that represent geometric SD factors of 2.6, 6.8, and 4.8. Three DRV data points with fold change in EC_50_ values > 615 and five ATV data points with fold changes in EC_50_ values > 100 are included in the figure and in the calculation of geometric mean with values of 615, 101, or 133, respectively. Dashed line represents the drug resistance cutoff used herein, with fractional values denoting the proportion of total isolates with loss of drug susceptibility above this cutoff. (**C**) Proportion of HIV-1_HXB2D_ infected MT-2 cultures with observable cytopathic effect after 35 days of selection in the presence of fixed concentrations of GS-9770 or ATV. (**D**) Progression of GS-9770 dose escalation resistance selections in MT-2 cells infected with HIV-1 IIIb strain. Passages with emergent viral variants encoding new amino acid substitutions in PR are indicated in red.

To determine whether HIV from other clades and subtypes were also susceptible to GS-9770, we tested its antiviral potency against a panel of 22 HIV clinical isolates in phytohemagglutinin (PHA)-stimulated human peripheral blood mononuclear cells (PBMCs). In all, 20 HIV-1 isolates were tested, including representative strains from group M (subtypes A, B, C, D, E, F, and G), group N, group O, and circulating recombinant forms (CRF01_AE, CRF02_AG), as well as two HIV-2 isolates. Following a 7-day infection, virus production was determined by measuring reverse transcriptase (RT) activity in cell-free culture supernatants. GS-9770 maintained high antiviral potency against all isolates tested, with the mean EC_50_ values of 7.3 nM (range of 1.9 to 26 nM) against HIV-1 and 26 nM against HIV-2 (Fig. 3A). In comparison, the mean EC_50_ values for the control PI DRV against these same isolates were 3.2 nM (range of 0.8 to 9.2 nM) for HIV-1 and 8.5 nM for HIV-2.

### Activity of GS-9770 against clinical HIV-1 isolates with resistance to other protease inhibitors

To better understand the antiviral activity of GS-9770 against HIV-1 with resistance to current PIs, GS-9770 was tested against a panel of 49 reporter viruses encoding primary and secondary PI resistance-associated mutations from PI-experienced PLWH (Tables S5 and S6). Whereas GS-9770 demonstrated potent antiviral activity against all clinical isolates tested, with only three viruses exhibiting a greater than 10-fold loss in susceptibility to this compound, 17 and 16 of these same isolates showed a greater than 10-fold loss in susceptibility to the control PIs DRV and ATV, respectively (Fig. 3B; Table S6). The mean fold change (FC) in the EC_50_ for GS-9770, DRV, and ATV against these mutants versus wild-type HIV-1 was 2.5, 5.3, and 9.2, respectively, and ranged from 0.4 to 21 for GS-9770, 0.3 to >615 for DRV, and 0.6 to >132 for ATV.

### Selection and phenotypic characterization of HIV-1 variants resistant to GS-9770

To identify potential resistance mutations that might emerge in the presence of this drug, MT-2 cells were infected with HIV-1 IIIb at a high multiplicity of infection (MOI = 0.1) and then passaged for 35 days in culture in the presence of GS-9770 or ATV (control PI) at fixed drug concentrations equal to twofold, threefold, or fourfold their mean respective 95% effective concentration (EC_95_) values of 21.6 and 19.5 nM, respectively. No breakthrough virus emerged from any of the four replicate wells assessed for each of the three GS-9770 concentrations tested (Fig. 3C). In comparison, parallel selections with ATV resulted in the emergence of virus from all four wells at both twofold and threefold EC_95_ drug concentrations. The breakthrough virus from these ATV selections did not contain amino acid substitutions in PR suggesting a loss of antiviral activity at these drug concentrations rather than selection of resistant variants.

In an effort to develop and identify amino acid substitutions that confer resistance to GS-9770, a dose escalating resistance selection was performed by infecting MT-2 cells with HIV-1 IIIb at low MOI (0.01) and serially passaging cells with increasing GS-9770 concentrations over the course of 368 days (Fig. 3D). After 171 days of selection, virus emerged in the presence of 100 nM GS-9770 (25-fold its EC_50_) and encoded three amino acid substitutions in PR (M46L, A77I, and V82A). Continued culturing in the presence of 100 nM GS-9770 resulted in the emergence of an additional L24I substitution after 213 days of drug selection, followed thereafter by the addition of an I64V substitution 29 days later at 150 nM GS-9770 (~38 fold its EC_50_). At the conclusion of this 368-day dose escalation experiment, the GS-9770-selected virus was able to replicate in the presence of 1.75 µM GS-9770 (437-fold its EC_50_) and contained a total of five PR substitutions (L24I, M46L, I64V, A77I, and V82A) that were encoded by a single genome and present in 100% (17/17) of clones analyzed.

HIV-1 (LAI strain) variants encoding GS-9770-selected PR substitutions were generated by site-directed mutagenesis and then assayed for infectivity and drug resistance relative to wild-type virus (Table 3). Introduced PR substitutions had variable effects on virus infectivity, with the V82A and L24I M46L I64V A77I V82A variants exhibiting the lowest infectivity at 36% and 48% of wild type, respectively. When profiled for possible drug resistance, virus encoding the M46L A77I V82A and L24I M46L A77I V82A substitutions in HIV-1 protease showed only minor losses in susceptibility to GS-9770 with mean EC_50_ FC values of 2.7 and 1.9, respectively, and full susceptibility to two control PIs (ATV and DRV) and the control NNRTI efavirenz (EC_50_ FC values ranging from 0.3 to 1.2). A 5× PR mutant HIV-1 variant (L24I M46L I64V A77I V82A) encoding all PR amino acid substitutions detected at the end of the GS-9770 resistance selection showed a moderate 7.3-fold loss in susceptibility to GS-9770, as compared to a 2.1-, 0.5-, and 0.8-fold loss in susceptibility to ATV, DRV, and EFV, respectively.

**TABLE 3:** Characterization of site-directed HIV-1 PR mutants from GS-9770 resistance selections

HIV-1 PR genotype^*a*^	GS-9770 concentration (nM) at variant first emergence (Fold EC_50_)^*b*^	Infectivity (% WT)^*c*^	EC_50_ (nM) (fold-change relative to WT)^*d*,*e*^			
			GS-9770	ATV	DRV	EFV
Wild type (WT)	N/A	100	3.1 ± 1.6 (1.0)	5.7 ± 3.6 (1.0)	4.4 ± 2.4 (1.0)	1.3 ± 0.7 (1.0)
V82A	N/A	36 ± 5	8.2 ± 3.2 (2.7)	7.6 ± 3.3 (1.3)	3.5 ± 1.5 (0.8)	1.2 ± 0.6 (1.0)
M46L A77I V82A	100 (25)	113 ± 6	8.3 ± 1.8 (2.7)	6.9 ± 1.8 (1.2)	2.5 ± 0.8 (0.6)	1.0 ± 0.2 (0.8)
L24I M46L A77I V82A	100 (25)	122 ± 8	5.9 ± 3.2 (1.9)	4.2 ± 0.8 (0.7)	1.4 ± 0.2 (0.3)	1.1 ± 0.1 (0.9)
L24I M46L I64V A77I V82A	225 (56)	48 ± 4	22.4 ± 7.3 (7.3)	11.7 ± 1.4 (2.1)	2.1 ± 0.5 (0.5)	1.0 ± 0.2 (0.8)

^*a*^ Site-directed mutations introduced into HIV-1 protease (PR) sequence of HIV-1_LAI_.

^*b*^ Resistance selection was initiated as drug concentration equal to 1× the EC_50_ for GS-9770 (4 nM); N/A = not applicable; the 5× mutant variant (L24I M46L I64V A77I V82A) persisted until end of study (368 total days of selection), reaching 437-fold the EC_50_.

^*c*^ % infectivity values (mean ± standard deviation) obtained from three independent experiments performed in duplicate.

^*d*^ EC_50_ values (mean ± standard deviation) obtained from four independent experiments performed in triplicate. Mean fold-change values (in parentheses) are calculated from the ratio of EC_50_ of the site-directed mutant virus over the EC_50_ of WT HIV-1_LAI_.

^*e*^ ATV = atazanavir; DRV = darunavir; EFV = efavirenz.

### Activity of GS-9770 against HIV-1 mutant variants resistance to other drug classes

The antiviral activity of GS-9770 was measured using a 5-day cytopathic antiviral assay in MT-2 cells individually infected with a panel of HIV-1 mutants with resistance to agents from other drug classes. Consistent with its distinct mechanism of action as an investigational HIV PI, GS-9770 retained full antiviral activity against each of the mutant HIV-1 isolates tested with mean EC_50_ FC values ranging from 0.4 to 2.3 (Table S7). In comparison, the control NRTI emtricitabine (FTC), the NNRTI efavirenz (EFV), and the INSTI elvitegravir (EVG) showed significant potency loss against select viruses encoding resistance-associated mutations within their respective viral target protein, with mean FC in mutant versus wild-type EC_50_ values ranging from 3.5 to >141.

### Two-drug *in vitro* combination studies

The antiviral activity of GS-9770 in pairwise combination with agents from other antiretroviral classes was evaluated in MT-2 cells infected with HIV-1_IIIb_ and the combinatorial effects analyzed using the Prichard and Shipman method assessed with MacSynergy II software (11). Results of these *in vitro* combination assays were expressed as the mean combination volumes (μM^2^%) calculated at the 99% confidence level from a minimum of three independent experiments performed in triplicate (Table 4). Pairwise combinations of GS-9770 with the NRTIs tenofovir alafenamide (TAF) or FTC, the NNRTI rilpivirine (RPV), the INSTI bictegravir (BIC), or the capsid inhibitor lenacapavir (LEN) resulted in highly synergistic anti-HIV activity with mean synergy volumes ranging from 163 to 180 µM^2^%. No antagonism was observed for any of the two-drug combinations tested with mean antagonism values ranging from 0 to −11 µM^2^%. Controls for additivity (GS-9770 combined with itself) and antagonism (ribavirin combined with stavudine) were assessed in parallel and each produced the expected result.

**TABLE 4:** *In vitro* drug combination studies with GS-9770

Drug combined with GS-9770^*a*^	TAF	FTC	RPV	BIC	LEN	GS-9770
Synergy volume [µM^2^.%]^*b*^	166 ± 51	139 ± 24	184 ± 65	156 ± 49	201 ± 111	23 ± 14
Antagonism volume [µM^2^.%]^*b*^	−4 ± 7	−8 ± 11	0 ± 0	−11 ± 13	−0.6 ± 1.7	−3 ± 3
Combination effect	Highly synergistic	Highly synergistic	Highly synergistic	Highly synergistic	Highly synergistic	Additive

^*a*^ TAF, tenofovir alafenamide (NRTI); FTC, emtricitabine (NRTI); RPV, rilpivirine (NNRTI); BIC, bictegravir (INSTI); EVG, elvitegravir (INSTI); LEN, lenacapavir (capsid inhibitor).

^*b*^ Combination volumes calculated at 99% confidence interval. Data are mean (± s.d.) values from at least three independent experiments (n = 3 biological replicates each).

## DISCUSSION

In this report, we describe the discovery and characterization of GS-9770, a potent new HIV protease inhibitor designed around a novel iminohydantoin core with improved metabolic stability as compared to current peptidomimetic PIs. GS-9770 demonstrated potent and selective inhibitory activity *in vitro* against recombinant HIV-1 protease with a > 660 fold preference for this enzyme over six other aspartic proteases tested. In light of the fact that the iminohydantoin core of GS-9770 was found by X-ray crystallography to engage the same D25 residues in HIV PR that are present in all the off-target aspartic proteases tested, these data indicate that protease specificity can be attained through modification of the P1, P1′, P2, and P2′ drug moieties.

GS-9770 demonstrated improved metabolic stability in the presence of human microsomes when compared to ATV and DRV. In addition, the *in vivo* clearance and half-life values obtained for GS-9770 following its oral and intravenous administration to nonclinical species correlated well with *in vitro* predicted values obtained using liver microsome fractions. Allometric scaling using these pharmacokinetic data together with a predicted human plasma clearance value of 0.09 L/h/kg determined using a radiolabeled compound, GS-9770 is predicted to have a plasma half-life of ~18.5 hours in humans. For comparison, the plasma half-life for ATV and DRV following a single oral dose co-administered with a pharmacological boosting agent in humans is 8.6 and 15 hours, respectively. These data suggest that GS-9770 has the potential to be a once-daily oral PI for the treatment of HIV-1 infection in PLWH.

In cell culture, GS-9770 demonstrated potent antiviral activity comparable to atazanavir in HIV-1-infected MT-4 T-cells, CD4 +T-lymphocytes, and monocyte-derived macrophages. Whereas GS-9770 displayed 2.1- to >11-fold lower selectivity index values in these cell types (with a low value of 620) relative to that of the control PI atazanavir, the cytotoxicity of GS-9770 in these and multiple other human cell types tested was overall quite low, with mean CC_50_ values ranging from 4.7 to 34 µM across 15 different cell types. In addition to its activity against the laboratory-adapted HIV-1 strains used above, GS-9770 also demonstrated comparable antiviral potency against HIV-2 and various subtypes of wild-type HIV-1 in human PBMCs (EC_50_ ranged from 1.9 to 31 nM), with no selective activity observed against multiple non-HIV viruses tested.

GS-9770 largely maintained its antiviral activity against a panel of 49 PI-resistant clinical isolates as compared to complete loss of activity against 3 and 5 of these isolates for the FDA-recommended drugs ATV and DRV, respectively. Of the three viruses that demonstrated a > 10-fold loss in susceptibility to GS-9770, these isolates contained a total of four (L10I, M46L, I54V, and V82A in virus 43), five (L10F/L, D30N, I54V, N88D, and L90M in virus 29) and six (L10I, M46I, G48V, I50V, V82A, and L90M in virus 13) PI-resistant substitutions, resulting in mutant vs wild-type HIV EC_50_ FC values for GS-9770 of 11, 12, and 21, respectively. However, as Monogram isolates with remarkably similar PR substitutions such as virus 8 (M46I, I50V, I54V, V82A, and L90M), virus 11 (M46L, G48V, I54M, V82A, I84V, and L90M), and virus 42 (D30N and N88D) showed only low-level (3-fold) loss in susceptibility to GS-9770, these data could suggest that resistance to this PI drug may be governed more by specific combinations of amino acid substitutions in protease rather than specific driver mutations themselves.

Resistance selections performed for just over a year in the presence of escalating GS-9770 concentrations resulted in the virus being replicate in 1.75 µM GS-9770 (437-fold its EC_50_) and containing five PR substitutions (L24I, M46L, I64V, A77I, and V82A). A site-directed HIV-1 mutant encoding these five PR substitutions showed a 7.3-fold loss in susceptibility to GS-9770 with minor cross-resistance to ATV (2.1-fold) and hypersensitivity to DRV (0.5-fold). The M46L and V82A substitutions are non-polymorphic PI resistance-associated mutations and were commonly found either alone or in combination in 59% (29/49) of the PI-resistant clinical isolates profiled herein. The V82 residue is located within the inhibitor binding pocket and makes van der Waals contacts with GS-9770 in the P1 pocket near the phenyl-triazole moiety and with the difluoro group in the P1′ pocket. Structural modeling indicates that a valine (V) to alanine (A) substitution at position 82 of HIV-1 protease can be expected to reduce the contact surface area that GS-9770 makes in this binding pocket. Consistent with this notion, the V82A single PR mutant virus was found to confer a minor loss in susceptibility to GS-9770 (2.7-fold). Development of the V82A-resistant variant appears to come at the expense of reducing virus infectivity to 36% of wild type and may explain why a virus encoding the V82A mutation alone did not emerge during *in vitro* selection for GS-9770 resistance. M46 is located near the edge of the binding pocket, close to the flap residues, but does not make any direct contact with GS-9770. Mutations at position M46 have been predicted to change the flexibility and dynamics of the flap (12). The L24I, I64V, and A77I substitutions were not present in any of the clinical isolates tested herein; however, L24I has been observed in PLWH receiving IDV and LPV therapy, whereas the I64V and A77I substitutions are non-polymorphic mutations often associated with resistance to PIs (13). Interestingly, the addition of M46L and A77I to the V82A substitution was able to fully restore virus infectivity and was the first variant to emerge in our dose escalation resistance selections.

Two-drug combination studies were performed to evaluate GS-9770 activity in combination with drugs from other antiretroviral classes of inhibitors. Synergy was observed for all two-drug combinations tested with no evidence of antagonism observed when GS-9770 was combined pairwise with an NRTI (TAF or FTC), an NNRTI (RPV), an INSTI (BIC), or a capsid inhibitor (LEN) indicating GS-9770 has the potential to be used in combination with drugs from other classes of HIV antivirals for the treatment of HIV-1 infection.

In summary, GS-9770 is a novel, potent, and selective non-peptidomimetic inhibitor of HIV protease with improved metabolic stability relative to commonly prescribed PIs. GS-9770 demonstrates potent antiviral activity against a broad range of HIV types, including viruses resistant to other classes of antiretrovirals and patient-derived isolates completely resistant to currently recommended PIs. Although further development of GS-9770 was ultimately not pursued due to bronchoalveolar inflammation observed in the lungs of dogs after repeated oral administration with high doses of GS-9770, these data, combined with its favorable high genetic barrier to *in vitro* resistance emergence, illustrate the potential for GS-9770 to help inform future drug discovery efforts toward the development of new PIs suitable for once-daily oral dosing in the absence of PK enhancers.

## MATERIALS AND METHODS

### Compounds

GS-9770, atazanavir (ATV), darunavir (DRV), amprenavir (APV), bictegravir (BIC), elvitegravir (EVG), lenacapavir (LEN), tenofovir alafenamide (TAF), emtricitabine (FTC), efavirenz (EFV), stavudine (d4T), the RSV control inhibitor YM-53403, and the SARS CoV-2 control inhibitor remdesivir (RDV) were all synthesized at Gilead Sciences (Foster City, CA). Ribavirin (RBV) was purchased from Combi-Blocks (San Diego, CA). The protein synthesis inhibitor puromycin and the HIV-positive control azidothymidine (AZT) were purchased from Sigma-Aldrich (St. Louis, MO). The HBV control inhibitor ND-654 was purchased from Pharmaron (Beijing, China). The HCV protease inhibitor danoprevir was purchased from Selleck Chemicals (Houston, TX). All drug stocks were prepared in 100% dimethyl sulfoxide (DMSO) and stored frozen at −20°C.

### Protein crystallization

Recombinant HIV-1 protease (IIIb strain) was expressed and purified as described previously (14). For crystallization trials, PR was concentrated to 12 mg/mL in a final buffer containing 10 mM sodium acetate pH 6.0, 10% glycerol, and 1 mM DTT. GS-9770 was dissolved in DMSO to make a 10 mM stock solution and then mixed with PR for a final concentration of 400 µM. Crystals were obtained in 2.25 M ammonium acetate, 100 mM MES pH 6.5 by hanging drop vapor diffusion at 20°C. Crystals were introduced to a cryoprotectant solution containing 30% ethylene glycol in addition to the above concentrations of the mother liquor components. The crystals were then cooled in a bath of liquid nitrogen.

### Data collection, model building, and refinement

Crystal diffraction data were collected at The Advanced Light Source on BL5.0.1 at a temperature of 100 K and processed with XDS (15). The structure was determined by the molecular replacement method using the program Phenix (16) with PDB code 3EL1 as a search model. In addition, simulated annealing, energy minimization, and B-factor refinement were carried out in Phenix. Model building was performed with the molecular graphics program Coot (17).

### Cell lines

The MT-2 and MT-4 T cell lines were obtained from the HIV Reagent Program (Division of AIDS, NIAID, NIH, Germantown, MD) and maintained at 37°C in 5% CO_2_ at densities below 0.6 × 10^6^ cells/mL by passaging in RPMI-1640 cell culture medium supplemented with 10% heat-inactivated fetal bovine serum (FBS, Hyclone, Logan, UT) and 1% penicillin-streptomycin (Gibco) (complete RPMI medium). The CEM-NK^R^ CCR5 +Luc + infectivity indicator T-cell line expressing the firefly luciferase gene under transcriptional control of the HIV-2 long terminal repeat (LTR) was obtained from the NIH AIDS Reagent Program and cultured in complete RPMI medium supplemented with 0.8 mg/mL Geneticin (Thermo Fisher Scientific, Chino, CA). Cultures were passages twice per week to keep densities below 1 × 10^6^ cells/mL. HEK293T cells were obtained from the Gladstone Institute for Virology and Immunology and maintained in Dulbecco’s modified Eagle’s medium (DMEM) supplemented with 10% FBS and 1% penicillin-streptomycin-glutamine (Gibco) (complete DMEM). The human hepatoma Huh-7 cell line was obtained from ReBLikon GmbH (Mainz, Germany) and maintained in complete DMEM. The human hepatoblastoma cell line HepG2, human prostate carcinoma cell line PC-3, human epithelioma cell line HEp-2, and normal fetal lung-derived cell line MRC-5 were obtained from the American Type Culture Collection (Manassas, VA). Hep-2 cells were maintained in complete DMEM whereas PC-3 and HepG2 cells were adapted to grow in 0.2% galactose-containing, glucose-free complete DMEM. MRC-5 cells were maintained in Eagle’s Minimal Essential Medium (MEM) supplemented with 10% FBS and 1% penicillin-streptomycin.

### Primary cells

Human peripheral blood mononuclear cells (PBMCs) were isolated from fresh leukopaks obtained from consenting healthy volunteers participating in an Institute Review Board (IRB) approved donor program, negative for HIV-1, hepatitis B, and hepatitis C viral infection (AllCells, Inc., Alameda, CA) and cryopreserved for long-term liquid nitrogen storage at −80°C in 90% FBS and 10% DMSO at a density of 5 × 10^7^ cells/mL. The preparation and culturing of CD4 +T-lymphocytes and monocyte-derived macrophage cultures from PBMCs has been previously described (18). Prior to infection, PBMCs and CD4 +T-lymphocytes were stimulated by culturing in a complete RPMI medium supplemented with 1 µg/mL phytohemagglutinin (PHA; Sigma, St. Louis, MO) and 5 ng/mL recombinant human interleukin-2 (IL-2; Roche Diagnostics, Indianapolis, IN) for 48 hours at 37°C. Maintenance of cryopreserved PBMCs used in cytotoxicity assays has been described previously (18). Maintenance of primary human hepatocytes purchased from Invitrogen and cultured in William’s Medium E has been described previously (19).

### HIV-1

HIV-1_IIIb_ and HIV-1_BaL_ strains were obtained through the NIH HIV Reagent Program (contributed by Dr. Robert Gallo, and by Drs. Suzanne Gartner, Mikulas Popovic, and Robert Gallo, respectively) and were passaged in human MT-2 cells and PBMCs, respectively, prior to their use. Two HIV-2 isolates (CDC310319 and CBL-20) and 20 clinical HIV-1 isolates from the Southern Research virus collection were selected for susceptibility profiling: 15 isolates spanning group M, including subtype A (92UG031, 92UG037), subtype B (YU-2, 96TH_NP1538, 91US004, JR-CSF, and Ba-L), subtype C (92BR025, 98US_MSC5016), subtype D (92UG001, 98 UG_57128), subtype E (CMU02, CMU08), subtype F (93BR020), and subtype G (JV1083), one isolate each from groups N (YBF30) and O (BCF01), and two isolates each from circulating recombinant forms CRF01_AE (90TH_CM235) and CRF02_AG (01 CM_0 08BBY, 91DJ263). Recombinant HIV-1 strains encoding mutation(s) conferring resistance to NRTIs, NNRTIs, and INSTIs were prepared by transfecting pLAI-based or HXB2D-based infectious molecular clones into HEK293T cells, followed by virus amplification in MT-2 cells and harvesting of the cell supernatants as previously described (18, 20). Recombinant HIV-1 strains encoding mutation(s) that emerged upon dose-escalation selection with GS-9770 were similarly prepared by site-directed mutagenesis and transfection of pxxLAI-based infectious molecular clones into HEK293T cells following a protocol previously described (21).

### Enzymatic assays

For the HIV protease assay, recombinant HIV-1 protease expression, purification, and enzymatic assay using a fluorogenic readout were performed as previously described with some minor changes (22). Recombinant HIV protease (2.5 nM) and variable concentrations of test compounds were added to the reaction buffer. After a 20-minute pre-incubation, the enzymatic reaction was initiated by the addition of the fluorogenic substrate to a final concentration of 40 µM and a final volume of 100 µL. The reaction was measured over 20 minutes on a Tecan Infinite M1000 plate reader using excitation and detection wavelengths of 320 nm and 420 nm, respectively. Reaction rates were plotted as a function of inhibitor concentration, and because of the high potency of the inhibitors, the data were fit using the program Dynafit (Biokin, Ltd.) to determine apparent inhibitory constant (Ki(app)) values.

A panel of off-target protease assays was performed to evaluate compound selectivity for HIV-1 protease. Except for the substrate used in the renin assay, all fluorogenic substrates and recombinant enzymes were purchased from R&D Systems and the assays were performed according to the manufacturer’s recommended procedure with minor changes. For the BACE1 assay, recombinant human BACE1 (50 nM) and test compound at various concentrations were added to a reaction buffer containing 20 mM sodium acetate at pH 4.8 with 0.06% Triton X-100 and 1% DMSO. After a 15-minute pre-incubation, the enzymatic reaction was initiated by the addition of fluorogenic substrate to a final concentration of 1 µM and a total volume of 100 µL. For the Cat D assay, after a 15-minute pre-incubation, the enzymatic reaction was initiated by the addition of the fluorogenic substrate to a final concentration of 4 µM and a total volume of 100 µL. For Cat E assays recombinant human Cat E was reconstituted to 2.4 µM in buffer containing 25 mM MES at pH 6.5 with 150 mM sodium chloride and activated according to the manufacturer’s instructions. Activated Cat E (0.5 nM) and test compound at various concentrations were added to the assay buffer containing 100 mM sodium acetate at pH 3.5 with 200 mM sodium chloride and 1% DMSO. After a 15-minute pre-incubation, the enzymatic reaction was initiated by the addition of the fluorogenic substrate to a final concentration of 20 µM and a total volume of 100 µL. For the pepsin assay, recombinant human pepsin (0.5 nM) and test compound at various concentrations were added to a reaction buffer with 1% DMSO. After a 15-minute pre-incubation, the enzymatic reaction was initiated by the addition of the fluorogenic substrate to a final concentration of 10 µM and a total volume of 100 µL. For the renin assay, renin was activated by mixing with equal volumes of trypsin and incubating for 60 minutes at 37°C. Activation was stopped with the addition of 3 µM aprotinin. Renin (25 nM) and test compound at various concentrations were added to the reaction buffer and after a 15-minute pre-incubation, the enzymatic reaction was initiated by the addition of the fluorogenic substrate DABCYL-GABA-Ile-His-Pro-Phe-His-Leu-Val-Ile-His-Thr-EDANS (AnaSpec, Inc) to a final concentration of 4 µM and a total volume of 100 µL. All off-target assays were measured for 10–15 minutes on a Tecan Infinite M1000 plate reader using an excitation wavelength of 320 nm and detection wavelength of 405 nm for the BACE1, Cat D, Cat E, and Pepsin assays or an excitation wavelength of 350 nm and detection wavelength of 490 nm for the renin assay.

Enzymatic assay data were analyzed in GraphPad Prism (version 9.3.0) by curve fitting with a four-parameter logistic equation to determine inhibitor half-maximal inhibitory concentration (IC_50_) values. Because the non-HIV enzymatic IC_50_ values for GS-9770 are significantly higher than the enzyme concentrations used, the IC_50_ values are equal to their Ki(app) values (23).

### Immunoblotting

To evaluate the effect of GS-9770 on intracellular Gag polyprotein processing, HEK293T cells were transiently transfected with an envelope-deleted HIV-1 reporter construct (pKS13), cultured in the presence of fixed GS-9770 concentrations, cell lysates prepared 48 hours post-transfection, and subjected to Western immunoblot analysis using monoclonal antibodies against p24 (1:1,000 dilution, Invitrogen, Inc., Cat. #MA1-71516) and anti-tubulin (1:2,000 dilution, Cell Signaling Technology, Cat. #2125, clone 11H10) as previously described (24).

### Antiviral assays

The 5-day cytoprotection antiviral assays using MT-2 and MT-4 T-cell lines acutely infected with syncytia-inducing HIV-1 strains (IIIb, LAI, or HXB2D) were performed essentially as previously described but with minor changes to the latter (18). MT-4 cells were infected with HIV-1 IIIb strain at a multiplicity of infection (m.o.i) of 0.005 for 1 hour and the assay was modified to a 384-well format with each drug tested at 10 concentrations in quadruplicate. Positive (10 µM AZT) and negative (0.5% DMSO) controls were included in every assay plate to define 100% and 0% protection, respectively.

Antiviral assays performed in HIV-1_BaL_ infected CD4 +T-lymphocytes and monocyte-derived macrophages (MDMs) have been described previously (18). Cell-free supernatants derived from the CD4 +T cell and macrophage cultures were harvested 7- and 12-days post-infection, respectively, and the amount of HIV present was quantified by p24 antigen enzyme-linked immunosorbent assay (ELISA, Perkin Elmer, Waltham, MA) performed according to the manufacturer’s protocol.

Antiviral assays using fresh human PBMCs independently infected with a panel of clinical HIV-1 and HIV-2 isolates were performed by Southern Research as a contracted research study and assessed 7 days post-infection using reverse transcriptase (RT) endpoint assay. Antiviral assays using HIV-1 reporter viruses containing patient-derived sequences encoding all of PR and a portion of RT (amino acids 1–313) and conferring resistance to FDA-approved HIV PIs were performed by Monogram Biosciences Inc. (South San Francisco, CA) using their previously described Phenosense assay (25).

Anti-HIV-1 assays evaluating the *in vitro* antiviral effect of two-drug combinations were performed in MT-2 cells as previously described (26). Data were normalized to positive and negative controls in each plate and expressed as a percentage of protection from CPE-dependent cell death and analyzed with the MacSynergy II software. Combination data were analyzed at the 99% confidence level, with synergy/antagonism volumes defined as follows: high synergy (>100 µM^2^%), moderate synergy (50 to <100 uM^2^%), additivity (>−50 to <50 µM^2^%), and antagonism (<−50 µM^2^%).

Antiviral assays using appropriate cell lines infected with non-HIV viruses were also performed. The hepatitis C virus (HCV) 1b and 2a replicon antiviral assays have been previously described (27) using danoprevir as a positive control. The hepatitis B virus (HBV) surface antigen (HBsAg) antiviral assay was performed as previously described (28) using ND-654 as a positive control. The respiratory syncytial virus (RSV) A2 HEp-2 antiviral assay was performed as previously described (29) using YM-53403 as a positive control. The severe acute respiratory syndrome coronavirus 2 (SARS-CoV-2) antiviral assay was performed as described previously (30) using remdesivir as a positive control.

For all antiviral assays described above, half-maximal effective concentration (EC_50_) values were calculated from compound dose-response curves using Prism 9 software (version 9.3.0) and a four-parameter, nonlinear regression model.

### Cytotoxicity assays

Compound cytotoxicity assessment in MT-4 cells, primary CD4+ T lymphocytes, and MDMs was carried out using a protocol identical to the antiviral assay except uninfected cells were used and tested with higher concentration ranges of test compounds. Assessment of cytotoxicity in additional human cell lines (Gal-PC3, Gal-HepG2, HEp-2, Huh-7, MRC-5, HepG2-NTCP, Huh7-Lunet HCV 1b and 2a replicons, NHBE, A549-hACE2) and primary human PBMCs (resting and stimulated with PHA/IL-2) and hepatocytes have been described previously (19). For all cytotoxicity assays, the CC_50_ value for puromycin was determined as a positive internal control and to assess assay sensitivity in each cell type. The effect of tested compounds on cell viability was measured using a CellTiter-Glo readout, performed according to the manufacturer’s recommendations.

### GS-9770 resistance analysis

Viral breakthrough selections were conducted under conditions of fixed, constant drug concentrations over a period of 35 days in MT-2 cells acutely infected with a high MOI (0.1) of HIV-1_HXB2D_ as described previously (18). Final drug concentrations assessed were equal to twofold, threefold, and fourfold the EC_95_ value of 21.6 and 19.5 nM for GS-9770 and ATV, respectively. The EC_95_ values for GS-9770 and ATV were calculated from their corresponding MT-4/HIV-1_IIIb_ EC_50_ values using the EC_anything_ equation: EC_F_ = EC_50_ X (F/100 F)^1/*n*^, where F equals the percent response (e.g., 95) and *n* equals the Hill coefficient for GS-9770 (*n* = 3.0) and ATV (*n* = 3.3) determined in MT-4 cells. Dose-escalation selections for drug-resistant HIV-1 variants were performed in MT-2 cells infected with HIV-1_IIIb_ (MOI = 0.01) using 1.5-fold incremental increases in the GS-9770 concentration from an initial starting concentration of 4 nM (equal to its EC_50_ value in MT-2/HIV-1_IIIb_ cultures) as previously described (18). In both types of resistance selections, viruses that emerged in the presence of GS-9770 or ATV were genotyped by population sequencing. Briefly, total RNA from cell culture supernatants was isolated using the QiaAMP Viral RNA Mini Kit (Qiagen), a 647-bp fragment encoding HIV-1 protease was amplified by PCR with reverse transcription (RT-PCR) using the Qiagen OneStep RT-PCR Kit in combination with the primers 5′-CAACTCCCCCTCAGAAGCAG-3′ and 5′-CTGCGGGATGTGGTATTCCT-3′, and then RT-PCR products were sequenced by Elim Biopharmaceuticals, Inc. (Hayward, CA). To assess the linkage of all observed substitutions, PCR products encoding the entire Gag- and PR-encoding region were subcloned using the TOPO TA cloning kit (Invitrogen) and individual bacterial colonies subjected to PCR and the products sequenced. Protease and Gag-encoding sequences from selected HIV-1 variants were aligned using Sequencher software (Gene Codes, version 5.4.6) with that of the input virus and/or virus passaged in the absence of drug as an internal control for genetic drift that may have occurred during the selection process.

The resistance profile and infectivity of each GS-9770-selected PR variant were determined in MT-2 cells after introducing observed protease substitutions, alone and in combination, into a wild-type infectious HIV-1 molecular clone (LAI strain). For infectivity measurements, wild-type and site-directed mutant HIV-1 variants were serially diluted to generate 12-point, 3-fold dilution curves and samples overlayed in white opaque 96-well assay plates onto CEM-NK^R^ CCR5 +Luc + indicator cells in the presence of DEAE-dextran (10 µg/mL final concentration). After 72 hours, the relative infectivity of each well was calculated using the resulting linear range of luminescence signals from each mutant expressed as a percentage of the wild-type virus infectivity as previously described (24). For resistance analyses, wild-type and mutant LAI viruses were assessed in triplicate in a 96-well version of the 5-day MT-2 cytopathic assay (18) for their susceptibility to GS-9770 and three control antiretrovirals (DRV, ATV, and EFV).

### Metabolic stability of GS-9770 in human liver microsomes

The stability of test compounds upon incubation in human liver microsomal fractions was determined as previously described (24). The amount of parent drug remaining after 2, 12, 25, 45, and 65 minutes was determined by LC-MS/MS and fit to equation $C_{t}=C_{0}∙e^{-K∙t}$ where *C_t_* is the percent of parent remaining at time = t, *C_0_* is the percent of parent remaining at time = 0, *t* = time and *K* is the first order elimination rate constant. Drug half-life (T_1/2_) was determined using the equation $T_{1/2}=\frac{ln2}{K}$ where K is the elimination rate constant calculated above. Predicted hepatic clearance was determined by the well-stirred model previously described (31). The percent hepatic extraction ratio (E_h_%) is the predicted hepatic clearance divided by human hepatic blood flow (1.3 L/h/kg) times 100%.

### *In vivo* pharmacokinetics

Preclinical pharmacokinetic studies were performed in male Sprague–Dawley rats, beagle dogs, and cynomolgus monkeys (three animals per dosing group in each species) following federal and Institutional Animal Care and Use Committee (IACUC) guidelines as described previously (32). After intravenous (1 mg/kg) and oral (5 mg/kg) administration of GS-9770, plasma was isolated at designated timepoints post-dose (>10 samplings between 0 and 24 hours for each species) and the concentration of GS-9770 was determined by LC-MS/MS. The drug concentrations versus time data obtained post-dosing were analyzed by non-compartmental analysis with Phoenix WinNonlin software v6.4.0.768 (Certara, Princeton, NJ) to provide an estimation of the area under the plasma concentration versus time curve from 0 to 24 hours (AUC_0-24h_), the terminal half-life (t_½_), the rate of systemic plasma clearance (CL) in liters per hour per kilogram (L/h/kg), and the steady-state volume of distribution (V_ss_). The oral fraction absorbed (F%) was calculated using the formula:


$$\%F=\frac{\left(\;PO\;Dose\;AUC_{0-inf}\right)\;x\;\left(IV\;Dose\right)}{\left(IV\;Dose\;AUC_{0-inf}\right)\;x\;\left(PO\;Dose\right)}\times 100\%$$

## Data Availability

Primary data are available from the corresponding author upon reasonable request. The atomic coordinates and structure factors have been deposited into the Protein Data Bank under accession code 8VB1. The nucleotide sequences of the protease gene from no drug control and GS-9770-resistant selection viruses have been deposited in GenBank and assigned the accession numbers PP209551, PP209552, and PP2092553, respectively.

## SUPPLEMENTAL MATERIAL

Tables S1 to S7 aac.01373-23-s0001.docx All 7 supplemental tables referenced in the article.

ASM does not own the copyrights to Supplemental Material that may be linked to, or accessed through, an article. The authors have granted ASM a non-exclusive, world-wide license to publish the Supplemental Material files. Please contact the corresponding author directly for reuse.
