# Supplementary material for: Preclinical characterization of a non-peptidomimetic HIV protease inhibitor with improved metabolic stability
Source: Antimicrob Agents Chemother. 2024 Feb 21;68(4):e01373-23. doi: 10.1128/aac.01373-23 (PMC10989020; doi:10.1128/aac.01373-23)
Supplement: Tables S1 to S7 — All 7 supplemental tables referenced in the article. [file aac.01373-23-s0001.docx]

**Preclinical Characterization of a Non-peptidomimetic HIV Protease Inhibitor with Improved Metabolic Stability**

Andrew Mulato^a#^, Eric Lansdon^d^, Ron Aoyama^c^, Johannes Voigt^d,f^, Michael Lee^e^, Albert Liclican^e^, Gary Lee^e^, Eric Singer^a^, Brian Stafford^c^, Ruoyu Gong^e^, Bernard Murray^c^, Julie Chan^e^, Johnny Lee^e^, Yili Xu^e^, Shekeba Ahmadyar^e^, Ana Gonzalez^b^, Aesop Cho^b^, George J. Stepan^e^, Uli Schmitz^d^, Brian Schultz^e^, Bruno Marchand^e^, Boris Brumshtein^e^, Ruth Wang^e,h^, Helen Yu^e^, Tomas Cihlar^a^, Lianhong Xu^b,g^ and Stephen R. Yant^a^

^a^Department of Virology, Gilead Sciences, Foster City, California, USA

^b^Department of Medicinal Chemistry, Gilead Sciences, Foster City, California, USA

^c^Department of Drug Metabolism, Gilead Sciences, Foster City, California, USA

^d^Department of Structural Biology and Chemistry, Gilead Sciences, Foster City, California, USA

^e^Department of Discovery Sciences and Technology, Gilead Sciences, Foster City, California, USA

^f^Present Address: Biomea Fusion, Redwood City, California, USA

^g^Present Address: Brii Biosciences, San Mateo, California, USA

^h^Deceased

Supplemental Tables

**Table S1. Data collection and refinement statistics for GS-9770 X-ray structure**

|  | **GS-9770** |
| --- | --- |
| **Data Collection** |  |
| Wavelength (Å) | 0.977 |
| Space Group | *P*22_1_2_1_ |
| Unit Cell (a, b, c in Å) | 46.5, 57.0, 85.7 |
| Resolution (Å) | 50.0-1.30 (1.38-1.30) |
| No. of reflections | 333,174 (56,318) |
| No. unique | 46,163 (4,483) |
| *I/σ* | 16.8 (1.79) |
| CC (1/2) | 99.9 (72.2) |
| Completeness (%) | 99.7 (98.1) |
| **Refinement Statistics** |  |
| Resolution (Å) | 47.5-1.30 |
| No. reflections (F≥0) | 54,650 |
| *R*-factor^a^ | 0.200 |
| *R*-free^a^ | 0.220 |
| RMS bond lengths (Å) | 0.007 |
| RMS bond angles (°) | 0.89 |

Numbers in parentheses represent highest resolution shell.

^a^ *R*-factor and *R*-free = ∑||*F*_obs_| - |*F*_calc_||/∑|*F*_obs_| for 90% of recorded data (*R*-factor) or 10% of data (*R*-free).

**Table S2. Metabolic stability of GS-9770 in human liver microsomes**

| **Parameter** | **GS-9770** | **ATV** | **DRV** |
| --- | --- | --- | --- |
| E_h_% | 9 | 63 | 81 |
| T_½_ (minutes) | >395 | 24 | 7 |
| Predicted CL (L/h/kg) | 0.11 | 0.8 | 1.1 |

E_h_%, percent predicted hepatic extraction; T_½_, drug half-life in the presence of human liver microsomes; Predicted CL, predicted human clearance; ATV, atazanavir (PI); DRV, darunavir (PI)

**Table S3. GS-9770 does not demonstrate selective antiviral activity against multiple non-HIV viruses**

| **Virus^a^** | **Cell Line** | **CC_50_ (µM)^b^** | **EC_50_ (µM)^b^** | **SI^c^** |
| --- | --- | --- | --- | --- |
| HBV | HepG2-NTCP | 8.4 ± 2.0 | 11 ± 4 | 0.8 |
| HCV-1b | Huh7-Lunet replicon | 15.1 ± 2.1 | 10.8 ± 0.5 | 1.4 |
| HCV-2a | Huh7-Lunet replicon | 15.1 ± 3.0 | 10.4 ± 0.3 | 1.5 |
| RSV | NHBE | 4.7 ± 1.7 | 2.5 ± 0.6 | 1.9 |
| SARS-CoV-2 | A549-hACE2 | 7.8 ± 2.1 | 5.3 ± 1.9 | 1.5 |

^a^ Antiviral activity measured by: HBV HBsAg antiviral assay; HCV1b and HCV2a replicon system; RSV-Fluc reporter assay; SARS-CoV2-Nluc reporter assay.

^b^ Data are mean (± s.d.) values from at least three independent experiments (n=4 biological replicates each).

^c^ SI = selectivity index (CC_50_/EC_50_ ratio)

**Table S4. Cytotoxicity of GS-9770 in human cell lines and primary cells**

|  | **CC_50_ (µM)** | |
| --- | --- | --- |
|  | **GS-9770** | **Puromycin** |
| Human cell lines |  |  |
| Gal-PC3 | 15 ± 4 | 0.45 ± 0.17 |
| Gal-HepG2 | 13 ± 6 | 1.4 ± 0.8 |
| HEp-2 | 17 ± 2 | 0.60 ± 0.30 |
| Huh-7 | 17 ± 3 | 0.80 ± 0.70 |
| MRC-5 | 20 ± 6 | 0.31 ± 0.20 |
| Human primary cells |  |  |
| Hepatocytes | 34 ± 16 | 1.6 ± 0.2 |
| Unstimulated PBMCs | 9.1 ± 5.5 | 0.54 ± 0.21 |
| Stimulated PBMCs | 9.1 ± 0.6 | 0.80 ± 0.15 |

Data are mean (± s.d.) values from at least three independent experiments (n=4 biological replicates each). Puromycin was assayed in parallel as a positive control for cytotoxicity.

**Table S5. Resistance-associated amino acid substitutions for the PI class**

| **PI Resistance-Associated Mutations in HIV Protease^a^** | |
| --- | --- |
| Primary PI resistance associated substitutions | D30N, V32I, M46I/L, I47V/A, I50L/V, I54L/M/V, Q58E, T74P, L76V, V82A/F/L/S/T, N83D, I84V, N88S, L90M |
| Secondary PI resistance associated substitutions | L10F/I/R/V, V11I, K20M/R/T, L24I, L33F, M36I/L/V, K43T, F53L/Y, I54A/S/T, I62V, H69K/R, A71V/T, G73A/C/S/T, V77I, V82M, I85V, N88D, L89I/M/V |

^a^ Adapted from the 2022 International Antiviral Society-USA (IAS-USA) Guidelines lists (Wensing, 2022). HIV protease substitutions associated with reduced susceptibility to the PIs atazanavir, darunavir, lopinavir, tipranavir, fosamprenavir, indinavir, nelfinavir, and/or saquinavir

**Table S6. Susceptibility of PI-resistant HIV-1 reporter viruses to GS-9770, DRV and ATV**


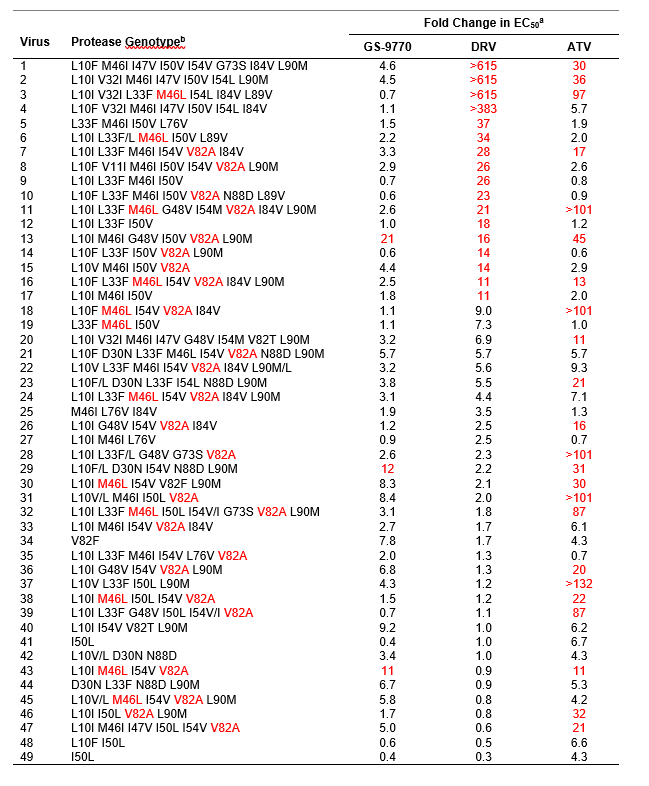


^a^ Fold resistance, HIV-1 mutant/wildtype EC_50_ ratio; red text represent changes in EC_50_ > 10-fold

^b^ PI resistance associated mutations that were also observed during the GS-9770 dose escalation study are displayed in red text

**Table S7. GS-9770 activity against HIV-1 isolates resistant to other drug classes**

| **Antiretroviral Class** | **HIV-1 Mutant** | **Fold resistance^a^** | | |
| --- | --- | --- | --- | --- |
|  |  | **GS-9770** | **Control Antiretroviral Agent^d^** | |
| Nucleotide reverse transcriptase inhibitor  (NRTI) | K65R | 1.5 ± 0.1 | FTC | 12 ± 6 |
|  | M184V | 2.0 ± 1.7 | FTC | >141 |
|  | 6TAMs^b^ | 2.3 ± 1.2 | FTC | 23 ± 21 |
| Nonnucleoside reverse transcriptase inhibitor  (NNRTI) | K103N | 0.6 ± 0.4 | EFV | 14 ± 12 |
|  | Y181C | 1.3 ± 0.1 | EFV | 3.5 ± 0.6 |
|  | Y188L | 0.9 ± 0.4 | EFV | >30 |
|  | L100I+K103N | 0.6 ± 0.3 | EFV | >30 |
|  | K103N+Y181C | 0.5 ± 0.2 | EFV | 11 ± 5 |
| Integrase strand transfer inhibitor (INSTI) | E138K+Q148K | 0.5 ± 0.2 | EVG | >50 |
|  | G140S+Q148R | 0.9 ± 0.2 | EVG | >50 |
|  | E92Q+N155H | 0.8 ± 0.2 | EVG | >50 |
|  | Q148+N155H | 0.4 ± 0.0 | EVG | >50 |

^a^ Fold resistance, HIV-1 mutant/wildtype EC_50_ ratio; 6TAMs, six non-polymorphic

^b^ HIV-1 reverse transcriptase mutations (M41L, D67N, K70R, L210W, T215Y and K219Q) that confer resistance to thymidine analogues.

^c^ Data are mean ± s.d. from 3 biological replicates in each of at least three independent experiments.

^d^ FTC, emtricitabine (NRTI); EFV, efavirenz (NNRTI); EVG, elvitegravir (INSTI).
